# Perceived Academic Team Leaders' Authentic Leadership and Team Members' Psychological Safety: A Cross-Sectional Online Survey

**DOI:** 10.1155/2024/5450333

**Published:** 2024-07-12

**Authors:** Majd T. Mrayyan

**Affiliations:** Department of Community and Mental Health Nursing Faculty of Nursing The Hashemite University, P.O. Box 330127, Zarqa 13133, Jordan

## Abstract

**Background:**

Current research has mainly concentrated on the psychological facets of authentic leadership and the sense of psychological security it cultivates.

**Aim:**

This research assessed the perceived academic team leaders' authentic leadership and team members' psychological safety.

**Methods:**

Using a quantitative cross-sectional study, the study was conducted in 2022 using an online survey. A convenience snowball sample of 105 nursing faculty members was recruited from various Jordanian universities.

**Results:**

The nursing faculty highly praised their leaders' authentic leadership on a 5-point scale. Yet, they felt a lack of psychological safety for themselves. While the academic nursing team leaders were commended for their readiness to hear others' suggestions before making choices, they should work on resisting group influence. These leaders must convey their feelings openly and truthfully. Concerning their own psychological safety, the nursing faculty felt their distinctive abilities and talents were recognized and utilized when collaborating with team members, which was the most highly rated feature. Conversely, the least-rated aspects were holding mistakes against faculty members and having trouble requesting assistance from others. The nursing faculty's sense of security and comfort significantly impacts their psychological wellbeing. Interestingly, their level of psychological safety is found to have a significant but negative correlation with their marital status, providing a rich and new insight into psychological safety; married females with children are prone to more work burnout, which might lower their psychological safety. On the other hand, a positive and moderate correlation is observed between psychological safety and the size of the team they work with. Surprisingly, the team size is the only factor that predicts the psychological safety of nursing faculty members; this occurs by enhancing the team's creativity and learning behaviors. However, the model itself is not very effective and only accounts for a small portion (6.30%) of the variation in their psychological safety scores, suggesting other unmeasured factors likely play a more significant role in nursing faculty members' psychological safety, such as personality traits, stressors, and job satisfaction.

**Conclusion:**

The authentic leadership displayed by nursing team leaders does not directly impact the psychological safety of nursing faculty members. The study addresses a critical and contemporary issue within the nursing academic field, providing useful preliminary insights. However, its methodological limitations, including sample selection and the weak explanatory power of its model, suggest that further research is needed. The results highlight the urgent need for immediate interventions to improve the chaotic academic environment they are currently facing, such as enhancing workplace friendship and authentic communication and using entrepreneurial and nonauthoritative leadership styles. Future studies could benefit from diverse samples, longitudinal design, and deeper analysis of contributing factors to psychological safety.

## 1. Introduction

Academic institutions rely on faculty members to generate and share knowledge, as well as foster a sense of creativity and innovation in their students and society [1]. To fulfill these responsibilities, faculty members must feel psychologically secure. The evolving nature of academic environments necessitates the cultivation of psychological safety among faculty members [1, 2]. However, this cannot be achieved overnight; it requires the presence of authentic leaders within academic teams who can establish a culture that encourages creativity and innovation [2–5].

In the realm of academic nursing and healthcare, a novel idea called authentic leadership has emerged [5]. Previous studies focused on the psychological aspects of psychological safety and authentic leadership [6–10], neglecting its application in nursing or academic environments. Several recent research studies have focused on the topic of authentic leadership and its relationship to the safety climate in nursing [4, 5, 11, 12]. These studies have also explored the psychological safety of staff members. However, research specifically examining the impact of authentic leadership on psychological safety was lacking. It is important to mention that authentic leadership is usually addressed from the perspectives of positive outcomes; however, it has negative outcomes as well, such as negative workplace behaviors such as bullying, incivility, and staff burnout [13]. In the current research, we assumed that authentic leadership has positive outcomes; yet, the exact reasons behind the positive outcomes of authentic leadership, such as commitment, trust, and engagement of employees in nonacademic organizations, remain uncertain [11, 13–21], which may be linked to the psychological wellbeing of nursing faculty members [22]. Employees experienced a heightened sense of purpose and fulfillment when guided by leaders who demonstrated authentic leadership. Yet, their day-to-day feelings of happiness and overall psychological safety remained largely unchanged [11, 13–21].

The leadership research has been critiqued for lacking contextual understanding; thus, this study would help faculty members and academic administrators understand the effects of authentic leadership on various outcomes, such as the current concept of faculty members' psychological safety [6–10]. As a senior faculty member and previous university administrator in Jordan, similar to other faculty members around the world, I attest that we are struggling with the consequences of the COVID-19 pandemic, such as our worsened financial burdens and the marginalized psychological safety, warranting immediate interventions as we are the role models for our current students who are the future nurses.

Authentic leadership is a leadership style demonstrated by individuals with strong moral principles who accept the consequences of their choices and base their judgments on lasting values rather than temporary gains [4, 5]. In today's ever-changing work environments, especially in academia, authentic leadership is a crucial aspect of individual and organizational behavior [3–5]. At an individual level, it refers to workers who uphold high integrity standards and have the potential to be authentic leaders [3–5]. On an organizational level, it is essential for leaders, particularly those in higher management, to embody authentic leadership [3–5]. Embracing authentic leadership, regardless of the level, promotes employees' psychological safety, which is defined as employees feeling comfortable sharing their thoughts, admitting errors, and being themselves at work without fear of negative consequences [6, 7]. There is a collective understanding that the workplace values openness, allows room for learning from mistakes, and accepts each individual's true self [6, 7]. In turn, psychological safety prevents harmful behaviors and negative consequences, like medical errors [3–5]. Professionals, including nursing faculty members, continuously strive for their rights and ideal work environments [3–5].

Academic nursing leaders' authentic leadership in Jordan was linked to knowledge sharing within the team and nursing faculty members' creativity [3]. Another study was conducted in Jordan to explore the humble leadership of academic leaders and its effect on the psychological safety of faculty [4]. Moreover, a comparative nursing study in Jordan explored the relationships between the concepts of authentic leadership and the safety climate. However, it did not specifically focus on the psychological safety of staff members [5]. The study found that military hospitals in Jordan had higher levels of authentic leadership among nurses and a generally positive perception of the safety climate. In contrast, government hospitals had a negative safety climate [5]. The current study is the first to link the perceived academic team leaders' authentic leadership and team members' psychological safety in Jordan.

### 1.1. Research Questions

To my knowledge, the topics investigated in nursing and academic nursing settings have never been the subject of a global investigation before this one. This study assessed nursing faculty members' perceptions of their academic team leaders' authentic leadership and their own psychological safety. The investigation at hand aimed to uncover the following: (1) how do nursing faculty members perceive the authentic leadership qualities of their academic nursing team leaders? (2) What are the perceptions of nursing faculty members regarding their own sense of psychological safety? (3) Does the perceived academic nursing team leaders' authentic leadership predict nursing faculty members' psychological safety? The current study's findings will be utilized to build leadership initiatives that uphold the authentic leadership of academic nursing leaders and establish inclusive work environments to enhance the psychological wellbeing of nursing faculty members.

### 1.2. Background

In the current dynamic work environment, it is essential to have leaders who create psychologically safe environments [3–7]. This is crucial for fostering creativity and adaptability at the individual, team, and organizational levels, even in academic settings [3]. The acquisition of authentic leadership skills is essential as it fosters a sense of psychological security within the workplace [7–10, 14], including academic settings. Due to the ongoing changes in our academic environments, which call for transformative leadership [4], this study has chosen to investigate authentic leadership instead of focusing on humble, autocratic, and democratic leadership [4]. Authentic leadership is essential for effective work environments and beneficial outcomes, including the psychological safety of the workforce [4–10, 14], especially for our academic faculty members at universities. Authentic leadership improves staff's self-efficacy, job satisfaction, retention, interdisciplinary teamwork [13, 15], work engagement [11, 16–21], organizational productivity, and citizenship [12, 21, 22]. In the clinical setting, authentic leadership results in high-quality and safe healthcare [4, 21] and safe medication practices [23]. Nonetheless, there is a lack of sufficient research, particularly when it comes to how authentic leadership helps in establishing psychological safety within the nursing profession [5].

Authentic leaders possess qualities that make them trustworthy, optimistic, ethically sound, skilled in navigating change, productive, and capable of assisting their team members [4, 11, 18, 19, 21, 22, 24–26]. In his book, Bill George [27] outlines essential behaviors for an authentic leader as (1) “purpose,” which facilitates the opportunity for passion, (2) “values,” which facilitate behaviors that are aligned to values, (3) “relationships,” which facilitate the importance of building strong relationships with team, (4) “self-discipline,” which facilitates consistency in good and hard situations, and (5) “heart,” which facilitates the benefit for displaying compassion and empathy for team's wellbeing. Moreover, authentic leaders have the following traits: (1) “self-awareness” includes accepting one's talents and weaknesses and showing tolerance for how they may influence others, (2) “balanced processing” involves examining pertinent information from different perspectives before making decisions, (3) “relational transparency” involves expressing emotions or encouraging others to do so to foster trust, and (4) “internalized moral” refers to acting following one's moral principles [4, 17, 21, 22, 24–26, 28–32].

Because psychological safety is a part of the safety climate [3–5, 11, 12], a positive team climate is a critical driver of psychological safety and would occur when leaders demonstrate supportive behaviors [3]. A psychologically safe work environment, including academic settings, is a product of positive leadership styles, such as transformational leadership and authentic leadership [33, 34] and professional nursing practices [33]. Authentic leaders have the power to foster a sense of psychological safety among their teams by promoting the right attitudes and behaviors. To establish a psychologically safe environment for faculty members in academic nursing, authentic leaders can empower other potential leaders within the team and ensure that the necessary behaviors are reinforced [3].

Creating conducive work atmospheres and establishing clear boundaries are essential for nurturing the mental wellbeing of nursing faculty members [35]. These studies highlight the importance of leadership actions that enhance psychological safety, including recognizing and utilizing the individual strengths and abilities of faculty members, refraining from holding past errors against them, and fostering a collaborative environment where seeking assistance from team members is encouraged [7, 10].

In Jordan, Elrehail et al. [2] conducted a study in universities to measure the effect of transformational and authentic leadership on innovation. Even though it was not about psychological safety or academic nursing settings, Elrehail et al. [2] found that authentic leadership did not have an impact on innovation. In contrast, transformational leadership and innovation were positively correlated. Contrary to Elrehail et al. [2], Alzghoul et al. [36] found that authentic leadership positively impacts the work environment, creativity, and productivity in Jordanian telecommunication companies. The work environment's climate mediates the relationship among authentic leadership, creativity, and job performance [36].

To sum up, when academic nursing leaders wholeheartedly embrace authentic leadership, there is a greater chance that nursing faculty members will feel psychologically secure. If faculty members do not feel psychologically safe, the long-term viability of academic settings is in jeopardy. Faculty members are increasingly mindful of the kind of work environments they desire, as well as their role in educating students.

## 2. Methods

### 2.1. Design

An online survey was conducted using a quantitative cross-sectional approach. While not incorporating experiments, cross-sectional surveys were employed to examine different environments and choose participants based on the topics of interest and their outcomes. This design is generally straightforward to execute and cost-effective, and it provides initial insights to guide the development of more sophisticated future research [37]. Moreover, unlike other observational approaches, such as longitudinal designs, cross-sectional studies do not track individuals over an extended period [37]. Nevertheless, it is important to note that the cross-sectional design cannot establish causation between variables [37].

### 2.2. Participants and Settings

In Jordan, there are a total of 12 undergraduate nursing programs, each led by the dean. Assisting the dean in their academic duties are the vice dean as well as the assistant dean for student affairs, the assistant dean for quality management, and the heads of the departments. It is worth noting that informal leaders also play a role in these programs.

The study's general population comprised Jordanian nursing faculty members, with the target population being those from various universities. The accessible population consisted of nursing faculty members from the selected universities. To gather data, the researcher utilized a nonprobability convenience snowball sampling technique was used. Convenience sampling is a nonprobability method through which individuals are selected based on their availability at the time of data collection. Snowball sampling is a nonprobability method in which the ongoing recruitment process depends on who the current participants already know. Both sampling techniques result in self-selection bias, preventing generalizations of the results to the whole population of participants. For instance, the nursing faculty members selected for the study were from the same university as the current researcher, which could introduce a self-selection bias. This means that individuals with specific characteristics may be more inclined to participate in the research compared to others.

Using the personal and schools' Facebook and own WhatsApp, a total of 105 nursing faculty members from two government and two private universities resulted in an 84.67% response rate, though this sample was not representative of the general population of nursing faculty members in Jordan and the target population of nursing faculty members in Jordanian universities. Despite the inability to determine the exact number of leaders or rating groups, this sample size of 105 was selected from a pool of 124 potential participants.

To ensure the statistical validity of the study, the well-known Thorndike's formula, *N* = 10(*k*) + 50, where *k* is the number of variables and 50 cases are added to account for the attrition rate, was employed to determine the necessary sample size [37]. The two major variables in this study were the perceived academic nursing team leaders' authentic leadership and the psychological safety of the nursing faculty members. Accordingly, *N* = 10(2) + 50, a minimum of 70 participants was required for this study [37]. However, a total of 105 nursing faculty members were successfully included in the study.

To accurately measure the psychological safety of nursing faculty members, the study required participants to meet certain criteria. It was required that they were actively working as nursing faculty members at a university's nursing school for a minimum of one year, as this would ensure a more precise evaluation of their leaders' authentic leadership, given that psychological safety tends to improve with increased work experience. Moreover, they were expected to show competence in utilizing technological tools to participate in the online survey.

### 2.3. Research Ethics

The study was authorized by the Institutional Review Board (IRB), where the author is currently working. The study was assigned the reference numbers 2/1/20/2021 (October 18, 2020) and 11/8/2021/2022 (July 25, 2022). Nursing faculty members were informed that their participation in the survey indicated their consent, and they had the option to skip the online survey if they wished. There were no conflicts of interest; however, selection bias is always accompanied by nonprobability sampling.

Ethical behavior and privacy protection are critical in online surveys. With online data collection, there is a need for robust consents that focus on future data sharing [37]. That is, there is a need to disclose the privacy policy. Since it is my own work, I described what personal demographics were collected and how the survey results will be shared. Data were kept anonymous using codes, and their email addresses were not collected in the dataset. Data were also kept confidential by securely storing data and ensuring that only the author had access. Moreover, faculty members were assured that the results were shared exclusively with nursing leaders at the specified universities in order to maintain confidentiality.

### 2.4. Data Collection

The survey had the following three sections: a 16-item authentic leadership perception assessment scale, a 7-item psychological safety scale, and nine academic sample characteristics. The survey was hosted on Google Forms from March 10th, 2022, for ten days, and it was designed to allow for one submission.

The researcher announced the study on her Facebook and WhatsApp, allowing participants to decide whether to take part and encouraging them to share the survey link with their friends and contacts. The current researcher began by introducing the study and allowing participants to choose whether they wanted to take part. The survey was then conducted online in English, which is the official language of nursing education. The online survey has detailed invitation letters, contact information, and a declaration of voluntary participation and consent to participate.

Before making it publicly available, I conducted a pilot run of the survey to check for its suitability and applicability of the scales in the Jordanian academic environments. The survey was sent by WhatsApp to ten colleagues, with no necessary changes needed. Once everything was in order, I shared the survey link on the Facebook pages of the Faculty of Nursing and my colleagues. Nursing faculty members who participated in the online survey were considered to have given their consent, and they were encouraged to invite others to take part as well. To ensure maximum participation, a reminder email was sent to nursing faculty members after five days, reminding them to complete the survey only once.

### 2.5. Instruments

Since the current sample consisted of nursing faculty members teaching in English, the tools were used in their original language. However, a preliminary study was conducted to test if the tools could be used effectively in the Jordanian academic environment, and the results were positive.

The authentic leadership perception assessment tool was the Authentic Leadership Questionnaire (ALQ) [31]. In October 2020, the Mind Garden Institute gave authorization to use the ALQ, which was copyrighted in 2007 by Avolio et al. [26]. The ALQ, a 16-item survey, utilizes a 5-point Likert scale to measure responses. These responses range from strongly disagree (1) to strongly agree (5). The ALQ consists of the following four different categories: self-awareness (items 1, 5, 9, and 13), an internalized moral (items 2, 6, 10, and 14), balanced processing (items 3, 7, 11, and 15), and relational transparency (items 4, 8, 12, and 16). These four dimensions are constantly reported in many recent research studies [4, 5], and meta-analytic and systematic reviews [13, 15] reported authentic leadership and psychological safety concepts. For instance, statements in the ALQ may include examples like leaders acknowledging their weaknesses or their actions align with their values.

To calculate scores, the results of relevant items are averaged, resulting in a total score and subscale scores. In terms of scoring, high authentic leadership is indicated by a score of four or higher on the Likert scale, while a score of three or below represents poor authentic leadership [38].

The ALQ has been utilized in different types of organizations and cultural settings. It has been employed to examine various aspects, including work environments [8, 14] and team performance [3]. The ALQ has been studied in organizations of different sizes and levels, and its psychometric model has been analyzed in multiple countries [23]. The original tool has predictive validity [31]. The reliability coefficient of the ALQ scale remains consistent with previous studies [4], at 0.95.

Edmondson [6] developed a scale consisting of seven statements to measure psychological safety. Participants were asked to rate their agreement on a scale from 1 to 5, ranging from strongly agree to strongly disagree. Some examples of statements included in the scale are “when I make a mistake on this team, it tends to be held against me” and “team members feel comfortable discussing problems and challenging issues.”

I utilized the scoring method developed by Sexton et al. [38] to assess authentic leadership. A score of four or higher on a 5-point scale indicated high psychological safety, while a score of three or below indicated poor psychological safety. The original tool has been found to have convergent validity [6]. In this study, the instrument's Cronbach's alpha was 0.71, slightly lower than the 0.76 reported in Wang et al.'s previous study [7].

The sample characteristics that were measured included gender (male and female), marital status (single married), age (≤34 years and >34 years), time commitment (full time and part time), level of education (Baccalaureate, Master's degree, or more), presence of official accreditation and quality initiatives in the employer organization (yes and no), tenure (indicating how long an employee has worked for an organization) (≤4 years, >4 years), team size (≤15 members and >15 members), and sector type (governmental and private). The inclusion of variables such as time commitment and team size was made based on the current researcher's perspectives on the concept of psychological safety.

### 2.6. Analyses of Data

Before commencing the analysis, the data underwent extensive data-cleaning procedures to eliminate any erroneous information. That is, online surveys are often associated with inaccuracies caused by respondents who rush through the survey and select the first response without considering its content, as well as those who intentionally provide nonsensical feedback; these responses should be eliminated. Furthermore, outliers were identified, and histograms were created to evaluate the data. It was concluded that there were no notable deviations present. Most of the questions in the online survey were formulated in a manner that motivated respondents to provide answers, thereby ensuring the absence of missing data [37].

The independent variables in this study were the characteristics of nursing faculty members and their perception of their academic nursing team leaders' authentic leadership. The dependent variable was the psychological safety of the nursing faculty members [37]. To answer the first and second research questions related to concepts measured in the current study, authentic leadership and psychological safety were treated as interval variables; thus, descriptive statistics, including means, standard errors of the means, medians, interquartile (IQR), range, and 95% confidence interval (CI) of the mean, were generated using SPSS version 25 [39]. The third research question assessed whether the perceived academic nursing team leaders' authentic leadership predicts nursing faculty members' psychological safety using the general linear model (GLM) with an alpha level of 0.05. The GLM is an extension of linear regression that can be used with a wide range of types of data while allowing for flexible modeling choices and diverse responses, resulting in robust predictions [37]. Prior to running the GLM, the assumptions of normality, linearity, sphericity, and independence associated with a linear regression model were assessed, and no significant deviations were observed [37].

## 3. Results

The majority of the nursing faculty members consisted of married women (*N* = 72, 68.60%; *N* = 81, 77.10%), ranging from young to middle aged as they aged less than 34 years (*N* = 87, 82.90%). Nursing faculty held Master's degrees or higher (*N* = 67, 63.80%) and were employed full time (*N* = 98, 93.30%) in governmental universities (*N* = 80, 76.20%) that were accredited (*N* = 99, 94.30%) and focused on quality improvement (*N* = 97, 92.40%). These faculty members had an above-average tenure of four years or more (*N* = 73, 69.50%) and worked in teams of varying sizes, with over fifteen members on average (*N* = 55, 52.40%). However, the ideal collaborative team size is typically between four and eight members (Table 1). The sample characteristics are similar to those of nursing faculty members working in higher education organizations in Jordan.

### 3.1. Perceived Team Leader's Authentic Leadership

Nursing faculty members in the study generally rated their academic nursing team leaders as having high levels of authentic leadership (based on the scoring of Sexton et al., 2006) [38]. For the whole sample, the nursing faculty members rated high (agreed) the authentic leadership of their academic nursing team leaders (mean = 3.65 and mean SE = 0.08). In academics, authentic nursing leaders were perceived to listen to the ideas of others before making decisions (mean = 3.90 and mean SE = 0.10). On the other hand, authentic nursing leaders need to learn how not to allow the group to pressure them (mean = 3.45 and mean SE = 0.11). They should also learn to openly share their feelings with others (mean = 3.46 and mean SE = 0.11) (Table 2).

### 3.2. Perceived Team Members' Psychological Safety

Based on the scoring of the study in [38], high psychological safety scored four or higher, while poor psychological safety scored three or below. For the whole sample, the nursing faculty members rated their psychological safety poorly (mean = 3.10 and mean SE = 0.06). The highest-rated nursing faculty members' psychological safety item was that they felt their unique skills and talents were valued and utilized when working with team members (mean = 3.54 and mean SE = 0.09). However, the lowest-rated nursing faculty members' psychological safety items were holding mistakes against the faculty member (mean = 2.97 and mean SE = 0.10) and having difficulty asking others for help (mean = 2.97 and mean SE = 0.11) (Table 3).

### 3.3. Predictors of Perceived Team Members' Psychological Safety

Before addressing the third research inquiry, correlations were documented. The psychological safety of nursing faculty members demonstrated a significant, unfavorable, and moderate correlation with their marital status (*r* = −0.204 at an alpha of 0.05). In addition, it exhibited a significant, favorable, and moderate correlation with the size of the team (*r* = 0.255 at an alpha of 0.01).

The GLM indicated that the perceived authentic leadership of academic nursing team leaders did not influence the psychological safety of nursing faculty members. The size of the team was the only factor that predicted the psychological safety of nursing faculty members (*B* = 2.044 and *p* value = 0.040). The model was not significant (*F*(*df* = 12) = 1.57, *p* value = 0.112, and *R*^2^ = 0.171, Table 4), and it explained only 6.30% of the variance in the mean score of nursing faculty members' psychological safety. This result suggests that other unmeasured factors likely have a more significant role in promoting nursing faculty members' psychological safety.

## 4. Discussion

This research examined the variables and predictors that influence how nursing team leaders are seen as authentic leaders by their faculty members and how this affects the psychological safety of the nursing faculty members. Surprisingly, the perceived authentic leadership of academic nursing team leaders did not have an impact on the psychological safety of nursing faculty members, which goes against what I had anticipated.

### 4.1. Perceived Team Leader's Authentic Leadership

Nursing faculty members perceived their academic nursing team leaders as highly authentic; this high rating of leaders is similar to that of Hassan and Din [1] and Lee et al. [11]. Authentic leadership should be consistently demonstrated, as it is associated with numerous beneficial outcomes for employees, such as increased job satisfaction and commitment [21]. These outcomes may also extend to nursing faculty members.

The most crucial quality displayed by academic nursing team leaders who are viewed as genuine is their willingness to listen to others' ideas before making decisions, similar to Alzghoul et al. [36]. This attentive listening cultivates a participatory approach to decision-making, empowering faculty members to contribute their innovative thoughts [1, 40], which in turn cultivates a feeling of psychological security [41]. This collaborative decision-making process also nurtures a constructive team dynamic grounded in mutual trust and psychological safety [40, 41].

The lowest mean of perceived academic nursing team leaders' authentic leadership was that the leader did not allow the group to pressure them. In the academic nursing landscape, team leaders often face the challenge of maintaining their authentic leadership. A key aspect is their ability to resist undue pressure from the group they lead. Navigating the hectic environment of academia, all academic leaders must shoulder significant responsibilities in managing their own stress and conflicts, as well as those experienced by their team members. Thus, it is crucial to prevent burnout among both leaders and faculty, a factor that research has shown can mediate the influence of authentic leadership on nurses' decisions to leave their roles [11, 19, 20]. Preventing burnout is particularly relevant for nursing academics.

Regrettably, our nursing team leaders in academia refrained from openly expressing their emotions to others. This type of behavior should be reduced and avoided in order to improve academic outcomes. However, it is natural for people to reveal only emotions that match their resolve, as supported by Purwanto et al. [42]. This is an essential component of one's psychological wellbeing [42].

### 4.2. Perceived Team Members' Psychological Safety

Nursing faculty members reported experiencing a concerning lack of psychological safety, consistent with findings from previous studies [43]. Psychological safety is a critical factor for both employee wellbeing and organizational success [43], so this deficiency is problematic. Our academic leaders must, therefore, strive to cultivate an environment of trust and security where nursing faculty can thrive and achieve new heights. Building this foundation of trust relies on authentic engagement and decision-making within the academic nursing realm. Therefore, authentic leaders should resist external pressures and encourage open expression of thoughts and emotions. Ideally, academic leaders will be reliable and dedicated to the welfare of their staff [43], such as the nursing faculty members in our case. When faculty feel their unique capabilities are valued and utilized by their team, they experience a sense of psychological safety [3, 6], which allows them to fulfill their essential roles in teaching, research, and management and to contribute innovative and creative ideas, akin to Abu Rabia [35].

In contrast, nursing faculty members felt uneasy because their superiors in the academic nursing world would use their mistakes against them. They also faced difficulties in getting help from others. These results indicate a lack of trust and teamwork in the academic nursing field. When there is no trust, people hide their thoughts and avoid admitting their errors, fearing consequences [43]. However, when professors feel safe, they are more likely to address their wrongdoings openly. Trust is the essential basis for building a secure and encouraging environment.

### 4.3. Predictors of Perceived Team Members' Psychological Safety

Correlations indicated that the psychological wellbeing of nursing faculty members was significantly, negatively, and moderately linked to their marital status. This discovery is noteworthy and original. This result contradicts Xia et al. [44], who reported no differences in the self-psychological safety maintenance of nurses during the COVID-19 pandemic based on the marital status. Given that most of our participants were married women who work as nursing faculty members, we could cautiously infer that single female nursing academics may experience a higher level of psychological safety. This is because married women, particularly those in troubled marriages, are more prone to psychological issues that have a detrimental impact on their sense of safety. It is worth noting that working women in Jordan face significant financial burdens due to shared household responsibilities.

In addition, female nurses bear the primary responsibility for managing their families, particularly when it comes to raising children. The cultural norms largely influence this situation in the Arab region, where men have limited involvement in household tasks. This result is consistent with Cañadas-De la Fuente et al. [45], who reported that married females with children are prone to more work burnout, which is expected to lower their psychological safety, which may apply to nursing faculty members.

The psychological safety of nursing faculty members correlated with the team size, supported by Albritton et al. [46]. They revealed a minimum team size of 9-10, close to the current team size, at which team psychological safety and learning behaviors become critical for team effectiveness. Effective team size promotes a sense of psychological safety at the team level. In turn, a team's belief in a safe and trusted environment is promoted, implying more brainstorming, critical thinking, effective problem solving and decision-making, better job performance, and creativity [36].

Per predictors, contradictory to my expectations, the GLM indicated that the perceived academic nursing team leaders' authentic leadership did not predict nursing faculty members' psychological safety (contradictory to Anugerah et al. [47] and Maximo et al. [48]). This result is consistent with the results of a study conducted in Jordan about academic nursing team leaders' authentic leadership and its relation with knowledge sharing among the team and nursing faculty members' creativity [3]. Authentic leadership did not predict knowledge sharing within the team or nursing faculty members' creativity [3]. Moreover, Chaudhary and Panda [49] reported that psychological safety failed to transfer the impact of authentic leadership on work engagement and creativity. Even though the prediction model was insignificant, which is believed to be related to confounding variables, the current researcher still strongly believes in the roles of authentic leaders in their team members' psychological safety. The insignificant model calls for studying noninvestigated variables that may have a role in promoting faculty members' psychological safety, such as individual personality traits, job satisfaction, or external stressors. More specifically, personality traits are an individual's distinctive character or qualities over time. As the healthcare and educational environment is complex and customer centered, multiple competing stressors, such as workload, shortage of staff, and time pressures, result in many negative consequences, such as job satisfaction and turnover [50]. The authors reported that the personality of the staff, including faculty members, will likely play a significant role in team dynamics, especially particularly when they are under pressure. Staff responses to stressors and, in turn, their job satisfaction are influenced by their personalities [50], regardless of the type of stressors and their magnitudes.

The study findings suggest that nursing faculty members' perceptions of their team leaders' authentic leadership did not significantly influence their sense of psychological safety. This result aligns with Elrehail et al.'s [2] observation that authentic leadership had no impact on innovation within Jordanian universities, which may also apply to psychological safety, as it is closely linked to creativity and innovation. However, this finding differs from the conclusions of Alilyyani et al. [13] in Saudi Arabia, who found that authentic leadership positively and directly influenced team effectiveness, nurses' work engagement, and psychological safety. Obviously, authentic leadership and psychological safety are presented in their unique organizational, cultural, and county contexts.

Accounting for the county context, for example, given the significant changes taking place in higher education institutions, particularly in terms of quality and accreditation efforts on national and international levels, academic nursing settings in Jordan are being influenced by various forms of leadership. These alternative leadership styles, such as entrepreneurial and nonauthoritative leadership, have an impact on nursing academics [51], along with energizing workplace friendship and authentic communication. In addition, leadership that prioritizes knowledge and fosters innovation in academic settings is crucial, as is supportive leadership [11, 19, 20, 52] that encourages creativity [11, 19, 20, 51, 53]. These different leadership approaches are necessary in the ever-changing and unpredictable academic work environment.

Considering the unique organizational context, the size of the team was the only factor that affected the psychological safety of nursing faculty members although the impact was not significant. It is worth noting that a larger team could potentially help our nursing faculty members overcome their perceived lack of psychological safety. In such a scenario, they would be more inclined to support each other, especially when facing criticism for their mistakes. Albritton et al. [46] revealed a minimum team size of 9-10, close to the current team size, at which psychological safety becomes critical for teamwork. This result adds to the nursing authentic leadership literature; that is, the common demographic variables linked to nursing authentic leadership are age and experience. For example, Puni and Hilton reported that junior nurses perceive their work supervisors as authentic leaders [54].

### 4.4. Limitations and Implications for Research

Additional research is necessary to explore various aspects of the topic at hand. For instance, it would be beneficial to conduct further studies on how marital status affects the psychological wellbeing of nursing faculty members. It has been observed that single female faculty members tend to feel more secure in this regard. However, it is important to note that the data collected for this study were limited in scope, and as it was obtained from only two governmental and two private nursing schools, using the convenience snowball sampling method might not be representative of all nursing faculty members in Jordan.

Furthermore, the snowball inclusion technique employed in the study also restricts information regarding the number of individuals approached to participate, as well as the number of organizational units or managers involved. Moreover, while using convenience and snowball samples is practical, they are prone to selection biases and potential systematic errors, limiting their representativeness and generalization [37].

The use of cross-sectional design is a major limitation in the current research as it is difficult to establish cause-and-effect relationships. It is only a snapshot; thus, it cannot be used to analyze behavior over a period or establish long-term trends [37], such as those related to authentic leadership and psychological safety.

It is also worth mentioning that the findings may have limited applicability to other countries, as the data were collected specifically in Jordan. As such, it would be valuable to conduct further research in different cultural settings, as academic environments in Jordan, particularly within nursing, tend to be highly collectivist and hierarchical, with a significant power distance culture, as seen in previous studies [7, 8, 14].

Future research could benefit from a more diverse random sample and deeper analysis of psychological safety underlying factors. Furthermore, the researchers should consider alternative research designs, such as qualitative methods and longitudinal design, to overcome the limitations of the current cross-sectional design. In addition, there is a need to investigate different types of nursing leadership in relation to the psychological safety of nursing faculty members. Understanding why the authentic leadership of academic nursing team leaders did not support psychological safety is crucial. The insignificant prediction model mandates studying other nonstudied variables that could influence faculty members' psychological safety, such as job satisfaction and tenure staff commitment, individual personality traits, or external stressors. Mediation studies can provide insights into how the authentic leadership of academic nursing team leaders impacts the psychological safety of nursing faculty members. Furthermore, validation studies can explore additional dimensions of authentic leadership that are associated with psychological safety beyond self-awareness, internalized morals, balanced processing, and relational transparency [53].

### 4.5. Implications for Education and Practice

In Jordan, the academic nursing settings have a culture of high-power distance, which is hierarchical and collectivist. [7, 8, 14] Such professional hierarchy is a barrier to psychological safety; faculty members would have limited freedom to speak and be themselves, contrary to leaders in higher positions. This is consistent with our sample reporting of insecurity because their superiors in the academic nursing field would hold their errors against them. Our faculty members also faced challenges in seeking assistance from others. These behaviors demonstrate a deficiency in trust and collaboration fueled by the high-power distance environments, supported by Edmondson [6] and De Smet et al. [3].

Hierarchical and collectivist cultures contribute to negative outcomes, such as workplace bullying and incivility [8]. High power distance cultures have certain traits, such as limited access to information for those in authority, the use of power to establish social order, division of the work environment into classes with restrictions, and a focus on outcome rather than quality of treatment and relationships [8, 14]. To mitigate the impact of this culture in academic nursing settings, it is important to develop low power distance cultures, supporting our nursing faculty members reporting that they feel psychologically safe when their unique skills and talents are valued and utilized when working with team members, calling for authentic leadership behaviors.

Hierarchical and collectivist cultures should be transformed into low-power distance cultures. The latter can be achieved by promoting independence, minimizing inequality, considering the balance of power to prevent inequality, and viewing power as a means of accessibility that changes frequently [8, 14]. By creating low-power distance cultures, bullying and incivility can be reduced in academic work environments [8, 14]. In addition, academic nursing team leaders should learn authentic leadership, as it supports the psychological safety of their faculty members [7, 9, 10].

Starting by decreasing the hierarchical and collectivist cultures, it is essential to improve the psychological safety of our nursing faculty members; it is crucial to create positive working conditions in academic settings [35]. It is important to distinguish between healthy and dysfunctional work environments. By cultivating a healthy work environment, we can support the wellbeing and engagement of nursing faculty members, fostering a sense of trust and belonging [35].

The psychological safety among nursing faculty members can be enhanced through many leadership interventions [7, 10]. Called “practical enablers of psychological safety,” O'donovan and Mcauliffe [55] reported that assessing these enablers is the first step in developing and maintaining staff's psychological safety. In healthcare, these enablers were grouped according to the following five themes: priority for patient safety, improvement or learning orientation, support, familiarity with colleagues, status, hierarchy and inclusiveness, and individual differences [55]. In academic settings, these enablers could be an improvement or learning orientation, support, familiarity with colleagues, status, hierarchy inclusiveness, and individual differences.

The current study reveals that these faculty members feel a sense of appreciation and recognition for their abilities and contributions, similar to other studies [3, 6]. Authentic academic nursing team leaders must foster and maintain such leadership behaviors, reflecting improvement, support, and inclusiveness of psychological safety enablers. Conversely, nursing faculty members express concerns about their errors being used against them and find it challenging to seek assistance from others, demonstrating status and hierarchy psychological safety enablers [3, 55]. Authentic academic nursing team leaders should promptly address these instances of low-rated psychological safety, as they hinder the establishment of a supportive and secure environment, demonstrating the support, familiarity with colleagues' psychological safety enabler, and inclusiveness while addressing individual differences [6, 55].

## 5. Conclusion

The nursing faculty perceived their academic nursing team leaders to display strong authentic leadership, but they felt that their own psychological safety was lacking. Interestingly, the level of authentic leadership exhibited by the team leaders did not have any impact on the psychological safety of the faculty members. The only factor that predicted the psychological safety of the nursing faculty members was the size of the team they worked with. However, this model was ultimately deemed insignificant, stressing the need for further research related to some unstudied variables, such as individual personality traits, job satisfaction, or external stressors.

Authentic leadership initiatives are necessary to establish favorable working conditions for nursing faculty. It should be noted that the cultivation of psychological safety cannot be achieved in a short time.

Further investigation involving a diverse and extensive pool of participants should be conducted across various nations and cultures, utilizing different leadership styles and research methodologies, such as qualitative research, to enable a deeper understanding of the reasons why the perceived authentic leadership of academic nursing team leaders did not have a predictive effect on the psychological safety of nursing faculty members [56, 56].

## Figures and Tables

**Table 1:** Nursing faculty members' characteristics (*N* = 105).

Characteristics	*N* (%)
*Gender*	
Male	33 (31.40)
Female	72 (68.60)

*Marital status*	
Single	24 (22.90)
Married	81 (77.10)

*Age*	
≤34 years	18 (17.10)
>34 years	87 (82.90)

*Time commitment*	
Full-time work	98 (93.30)
Part-time work	7 (6.70)

*Level of education*	
Baccalaureate degree (clinical instructor)	38 (36.20)
Master's degree or above (faculty members)	67 (63.80)

*Accreditation initiatives in organizations*	
Yes	99 (94.30)
No	6 (5.70)

*Quality initiatives in organizations*	
Yes	97 (92.40)
No	8 (7.60)

*Number of tenures at work*	
≤4 years	32 (30.50)
>4 years	73 (69.50)

*Team size at work*	
≤15 members	55 (52.40)
>15 members	50 (47.60)

*The sector of work*	
Governmental	80 (76.20)
Private	25 (23.80)

**Table 2:** Nursing faculty members' perceptions of their academic nursing team leaders' authentic leadership (*N* = 105).

Items	Mean	Mean (SE)	Median	IQR 25^th^–75^th^	Range	95% confidence interval	
						Lower	Upper
(1) My leaders can list three of their greatest weaknesses	3.65	0.11	4	3–5	4	3.42	3.89
(2) My leaders' actions reflect their core values	3.71	0.11	4	3–5	4	3.48	3.94
(3) My leaders seek others' opinions before making up their own minds	3.70	0.11	4	3–5	4	3.47	3.93
(4) My leaders openly share their feelings with others	3.46	0.13	4	3–5	4	3.20	3.72
(5) My leaders can list three of their greatest strengths	3.79	0.11	4	3–5	4	3.56	4.02
(6) My leaders do not allow group pressure to control them	3.45	0.11	4	3–5	4	3.22	3.68
(7) My leaders listen closely to the ideas of those who disagree with them	3.66	0.10	4	3–5	4	3.46	3.87
(8) My leaders let others know who they truly are as persons	3.76	0.10	4	3–5	4	3.55	3.97
(9) My leaders seek feedback as a way of understanding who they really are as persons	3.79	0.10	4	3–5	4	3.59	3.99
(10) Other people know where my leaders stand on controversial issues	3.60	0.11	4	3–5	4	3.38	3.83
(11) My leaders do not emphasize their own points of view at the expense of others	3.60	0.10	4	3–5	4	3.40	3.81
(12) My leaders rarely present a “false” front to others	3.49	0.10	4	3–5	4	3.28	3.70
(13) My leaders accept the feelings they have about themselves	3.63	0.11	4	3–5	4	3.40	3.86
(14) My leaders' morals guide what they do as leaders	3.77	0.10	4	3–5	4	3.54	4.00
(15) My leaders listen very carefully to the ideas of others before making decisions	3.90	0.10	4	3–5	4	3.69	4.11
(16) My leaders admit their mistakes to others	3.49	0.12	4	3–5	4	3.24	3.74
Total score	57.95	1.37	60	52–68	64 (16–80)	55.22	60.68
Total mean score	3.65	0.08	3.80	3.35–3.80	4 (1–5)	3.48	3.82

This 16-item scale rated from 1 (strongly disagree) to 5 (strongly agree). SE = standard error of the mean; 95% confidence interval (CI) of the mean using standard errors.

**Table 3:** Nursing faculty members' perceptions of their own psychological safety variables (*N* = 105).

Items	Mean	Mean (SE)	Median	IQR	Range	95% confidence interval	
						Lower	Upper
(1) If I make a mistake on this team, it is often held against me	2.97	0.10	3	2–4	4	2.77	3.18
(2) Members of this team can bring up problems and tough issues	3.12	0.10	3	2–4	4	2.92	3.33
(3) People on this team sometimes reject others for being different	3.07	0.11	3	2–4	4	2.85	3.29
(4) It is safe to take a risk on this team	3.10	0.09	3	2–4	4	2.90	3.29
(5) It is difficult to ask other team members for help	2.79	0.11	3	2–4	4	2.56	3.02
(6) No one on this team would deliberately act in a way that undermined my efforts	3.11	0.10	3	2–4	4	2.92	3.31
(7) Working with members of this team, my unique skills and talents are valued and utilized	3.54	0.09	3	2–4	4	3.35	3.74
Total score	21.70	0.43	21	19–24	28 (7–35)	20.83	22.57
Total mean score	3.10	0.06	3	2.71–3.00	4 (1–5)	2.97	3.22

This 7-item scale rated from 1 (strongly disagree) to 5 (strongly agree). SE = standard error of the mean; 95% confidence interval (CI) of the mean using standard errors.

**Table 4:** The perceived academic nursing team leaders' authentic leadership and subject's characteristics as predictors of nursing faculty members' psychological safety using GLM (*N* = 105).

Dependent and significant predictors	*B* ^*∗*^	*T*-test	*P* value	*R* ^2^	Adjusted *R*^2^	*F*-test (*df*) ^*∗∗*^(*P* value)
The total score of nursing faculty members' psychological safety				0.171	0.063	1.57 (12) (0.112)
The total score of perceived leaders' authentic leadership	0.005	0.17	0.865			
Gender	−1.203	−1.18	0.239			
Marital status	−1.897	−1.66	0.100			
Age						
Time commitment	−0.212	−0.11	0.908			
Level of education						
Accreditation initiatives in organizations	1.877	0.95	0.343			
Quality initiatives in organizations	−1.465	−0.89	0.375			
Number of tenures at work	1.551	1.43	0.156			
Team size at work	2.044	0.98	0.040			
The sector of work	−0.926	−0.82	0.410			

^*∗*^ *B* = unstandardized coefficients; ^*∗∗*^*P* < 0.001 (2-tailed).

## Data Availability

The data used to support the findings of this study are available on request from the corresponding author. The data are not publicly available due to privacy or ethical restrictions.
